# Enhanced Lithium Storage in Hierarchically Porous Carbon Derived from Waste Tea Leaves

**DOI:** 10.1038/srep39099

**Published:** 2016-12-14

**Authors:** Changhoon Choi, Seung-Deok Seo, Byung-Kook Kim, Dong-Wan Kim

**Affiliations:** 1 School of Civil, Environmental and Architectural Engineering, Korea University, Seoul 136-713, Republic of Korea; 2 High-Temperature Energy Materials Research Center, Korea Institute of Science and Technology, Seoul 136-791, Republic of Korea

## Abstract

In this study, highly nanoporous carbon (HCl-TW-Car) was successfully synthesized using a facile procedure combining acid treatment with a carbonization process that uses waste tea leaves from spent tea bags as raw materials. The acid treatment not only promotes the efficient removal of unnecessary inorganic impurities but also increases the product porosity to enable synthesis of hierarchically porous carbon materials with various micro-, meso-, and macropores. When used as an anode material for lithium-ion batteries, HCl-TW-Car demonstrated a much higher discharge capacity than is theoretically possible using graphite [479 mAh g^−1^ after the 200^th^ cycle at a rate of 0.2C (1C = 372 mA g^−1^)] and exhibited greater rate capabilities compared with those of carbonated products from tea waste without acid treatment. It was shown that the good electrochemical properties of HCl-TW-Car can be ascribed to large Brunauer–Emmett–Teller (BET) surface area, well-formed hierarchical pores, and the prevention of unexpected electrochemical reactions from the reduction of metallic atoms.

Despite the continued depletion of fossil resources and severe increases in environmental problems including global warming and air pollution, the global energy supply continues to primarily rely on fossil fuels such as petroleum, coal, and natural gas. Critical problems of fossil fuel use include non-renewability, production of various greenhouse gases and pollutants, and unexpected price fluctuations. These problems have stimulated global interest in clean, sustainable, and renewable power sources such as solar, wind, and geothermal^1^,^2^,^3^, but such technologies still await the development of more effective energy storage systems^4^ and have high setup and maintenance costs.

Ever since lithium ion batteries (LIBs) were first commercialized by Sony Corporation in 1991, LIBs with high energy density, long life cycle, and power density have been the most suitable energy storage devices for hybrid electric vehicles (HEVs) and mobile electronic devices^5^. In addition, LIBs are more environmentally benign as energy storage systems than other secondary devices such as lead-acid or Ni-Cd batteries^6^,^7^. Research to discover appropriate anode materials is one of the more pressing issues in building the next generation high-performance LIBs. At present, graphite with the highest degree of graphitization is widely used as a commercial anode material for LIBs owing to its advantages of low cost, great cycle stability, high coulombic efficiency, and low electrochemical potential with respect to lithium metal^8^,^9^,^10^. However, the low theoretical capacity (372 mA h g^−1^) and very limited rate capability of commercial graphite cannot meet the ever-increasing demand for higher energies and more powerful LIBs for widespread use in applications such as HEVs, portable electronics, and large energy storage systems^11^,^12^.

Recently, numerous studies have been carried out to develop promising next-generation anode materials based on Si, Sn, Ge, and transition metal oxides^13^,^14^,^15^,^16^. However, these alternatives still have drawbacks compared with graphite, including large volumetric change, poor cycle stability, and large irreversible capacity. These critical problems must be solved to commercialize non-carbon-based anode materials with high electrochemical performance. For this reason, several newly developed carbon-based anode materials have been studied as candidates to substitute graphite in LIBs, including graphene nanosheets, carbon nanotubes, hollow carbon spheres, and carbon nanofibers^17^,^18^,^19^,^20^. However, complex manufacturing processes and the high cost of raw carbon precursors to synthesize these carbon materials have constrained mass production. As an available natural resource, biomass has recently attracted a great deal of attention for use in the preparation of carbon anode materials owing to ease of access, eco-friendliness, and low cost. Thus, the use in LIBs using carbon obtained from biomass waste has been explored with sources as diverse as rice husks, mangrove charcoal, wheat stalk, banana peels, cherry stones, and peanut shells^21^,^22^,^23^,^24^,^25^,^26^,^27^,^28^. The electrochemical properties of such materials are critically dependent on the optimization of the preprocessing procedure, pyrolysis temperature, and reaction time to control product crystallinity, morphology, and porosity^29^,^30^.

After water, various types of tea are the most consumed beverage worldwide, and several billion tons of tea are produced each year for consumption. In particular, over three-quarters of the tea brewed in the United States has been prepared using tea bags in recent years. The use of tea waste from tea bags for LIB anodes has rarely been investigated thus far.

In this study, we developed a low cost, simple process that combines hydrochloric acid treatment and carbonization for the preparation of hierarchically porous carbon anode materials derived from tea waste. Through comparison with a control experiment without acid treatment, we investigated the critical effects of the acid treatment used in this application. The electrochemical performance of carbon materials from tea waste as anode materials for LIBs was evaluated systematically. We demonstrated that this approach provides an effective preparation process for synthesizing efficient carbon materials from similar biomass raw materials for use in applications such as secondary batteries, supercapacitors, and catalysts.

## Results and Discussion

### Preparation of hierarchically porous carbon

**Figure 1:**
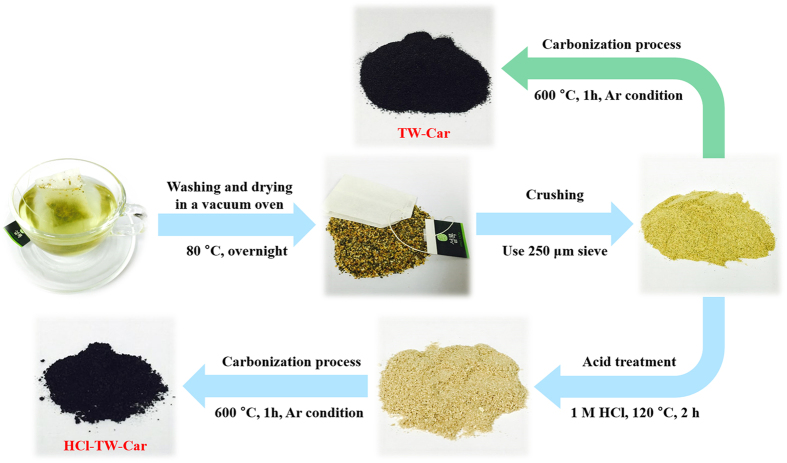
Synthesis process with/without acid treatment for the production of porous carbon derived from tea waste.

The synthesis strategy for porous carbon materials from tea waste was based on the form of the raw materials, the possibility for mass production, and the ease of transformation to a final product for LIB anodes. Figure 1 illustrates the key synthetic processes, including acid treatment and carbonization. The completely dried tea waste was first crushed using a mortar and pestle and then sifted with a 250 μm sieve. The acid treatment of the crushed powder was preferentially applied before carbonization. The final hierarchically porous carbon was denoted HCl-TW-Car. For comparison, simply carbonized samples (denoted TW-Car) were also prepared following the same carbonization process without acid treatment. In previous studies, acid treatment under various optimized conditions was used as a suitable pre-treatment before carbonization of the biomass^23^,^31^,^32^. In this study, the optimized condition for acid treatment was attained by controlling the reaction time and acid concentration. Using the optimized condition (1M HCl, 120 °C, 2 h), we obtained fine brown powders with a dramatic volume change in the materials, as shown Figure S1.

To determine the carbonization temperature, we conducted the thermogravimetric analysis (TGA) of the tea waste under exactly the same conditions as the carbonization process (Figure S2). The first weight loss observed around 100–200 °C was caused by the loss of superficial moisture and volatiles from the crushed tea waste. The major weight loss zone of these was between 200 and 350 °C, which can be attributed to the evaporation of volatiles upon decomposition of the various organics comprising the tea waste. The third weight loss, which continued beyond 350 °C up to 600 °C, was the result of complex carbonization reactions including aromatic condensation processes and evaporation of volatile vapors. An additional change that occurs at temperatures higher than 600 °C can be attributed to carbon layer formation.

From the perspective of economic feasibility and sustainability for widespread application, the yield of product and energy consumption of the synthetic process need to be considered. According to our experimental results, the typical yield of the carbonization process for synthesizing TW-Car was 24.8% as calculated based on the initially crushed tea waste, which corresponded with the total weight loss from the TGA data (72.3%) as well as the yield of carbon materials from various biomass types in the previous studies^25^,^31^,^33^,^34^. Moreover, the typical yield of HCl-TW-Car was estimated to be 20.0% of the raw materials (based on an acid treatment yield of 25.5% and a yield of the following carbonization process of 78.5%). Thus, our synthesis route represents a suitable approach for industrial production.

**Figure 2:**
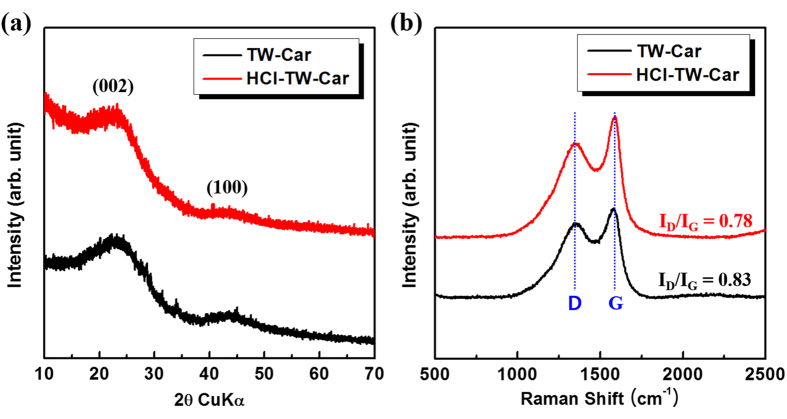
(**a**) XRD patterns and (**b**) Raman spectra of TW-Car (without acid treatment, carbonization at 600 °C for 1 h) and HCl-TW-Car (with acid treatment, carbonization at 600 °C for 1 h).

The crystalline structures of products obtained with and without acid treatment were obtained by X-ray powder diffraction (XRD) analysis, as shown in Fig. 2a. It is clearly seen that the XRD patterns of TW-Car and HCl-TW-Car possess no distinct characteristic peaks, indicating that both products demonstrate the typical structure of disordered carbon materials^35^,^36^. There are only two broad peaks at around 23° and 43°, which are assigned to the (002) and (100) crystallographic planes of graphene stacks, respectively. The broad (100) reflections at around 43° arose from honeycomb structures formed by sp^2^ hybridized carbons, while the broad (002) reflections at around 23° indicate small domains of coherent and parallel stacking of the graphene sheets^37^. Based on the peak positions of (002) reflections of both samples (TW-Car and HCl-TW-Car), they have the same calculated d-spacing value of 0.386 nm. Compared to the d-spacing value (0.335 nm) of a pure graphitic carbon structure, the observed larger d-spacing value of our products is beneficial for Li^+^ intercalation/deintercalation^38^.

The Raman spectra of synthesized products shown in Fig. 2b exhibit a similar trend to that of the XRD patterns. The two prominent peaks located at 1,349 and 1,580 cm^−1^ can be assigned to the well-documented D and G bands of carbon materials, respectively. The D band can originate from defects in the structure and disorder-induced features of carbon, while the G band can be attributed to the vibration of sp^2^ hybridized carbon^39^,^40^. Generally, the D/G intensity ratio of band intensities (I_D_/I_G_) is widely accepted as an appropriate index for the degree of structural disorder^41^,^42^. The I_D_/I_G_ ratio of TW-Car was determined to be 0.83, which is higher than that of HCl-TW-Car (I_D_/I_G_ = 0.78). The introduction of HCl pretreatment could lead to the removal of trace impurities. Although the crystallinity change is negligible by the removal of impurities as shown in the XRD patterns in Fig. 2a, the results of Raman analyses (Fig. 2b) confirm that HCl-TW-Car has a relatively lower degree of disorder and defects such as vacancies, dangling bonds, and topological defects than TW-Car, which could be related to the increase in the local stacking and ordering of the graphene-type layers. Based on these results, we could not easily determine whether TW-Car or HCl-TW-Car is a more suitable material for a LIB anode material; thus, we had to conduct a more comprehensive suitability test based on other properties (including morphology and porosity) and on the peculiarities of biomass (such as the presence of unexpected metallic atoms and impurities).

**Figure 3:**
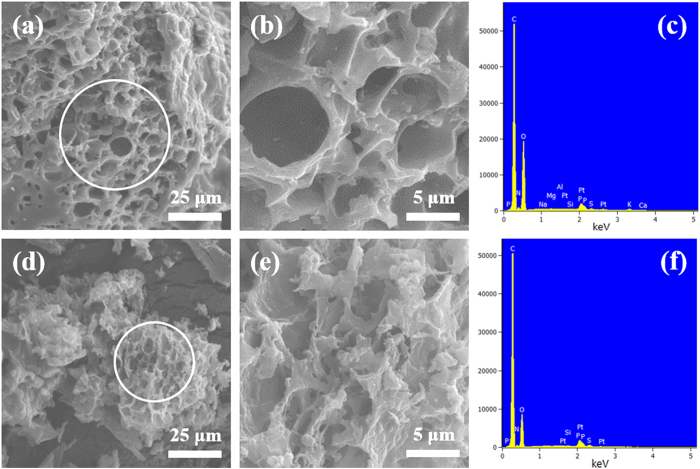
Low- to high-magnification SEM images of (**a,b**) TW-Car ((**b**): Zoomed-in image of the area circled in (**a**)) and (**d,e**) HCl-TW-Car ((**e**): Zoomed-in image of the area circled in (**d**)); EDS spectra of (**c**) TW-Car and (**f**) HCl-TW-Car from analysis of SEM images.

The field-emission scanning electron microscopy (FE-SEM) analysis was employed to analyze the microstructures of the samples. As raw materials, the washed tea waste possess irregular flake structure with sizes ranging from tens to hundreds of micrometers, as shown in Figure S3. In addition, the raw materials have no surface pores. On the other hand, both TW-Car (Fig. 3a,b) and HCl-TW-Car (Fig. 3d,e) exhibit sponge-like macroporous structures with sizes ranging from 3 to 10 μm; this is indicative of morphological transformation that occurs during the carbonization process and indicates that the optimized condition of the carbonization process is suitable for forming interconnected macropores within the carbon structures. Remarkably, there was no obvious change of morphology induced by the HCl acid treatment; instead, only a slight microstructure collapse occurred in the HCl-TW-Car, as shown in Fig. 3e. To confirm the effects of acid treatment, we conducted energy dispersive spectroscopy (EDS) analysis using the SEM images of both products. From Fig. 3c,f, and Table S1, it is seen that the content of metallic atoms (K, Ca, Mg, Al, and Na) disappeared as a result of acid treatment, which indicates the possibility of additional pore generation. Furthermore, X-ray photoelectron spectroscopy (XPS) was employed to further identify the nature of the functional groups. As observed in Figure S4, HCl-TW-Car showed lower proportion of C−O and C=O than TW-Car but higher proportion of functional nitrogen groups (especially, graphitic-N) than TW-car, which is consistent with the EDS analyses.

**Figure 4:**
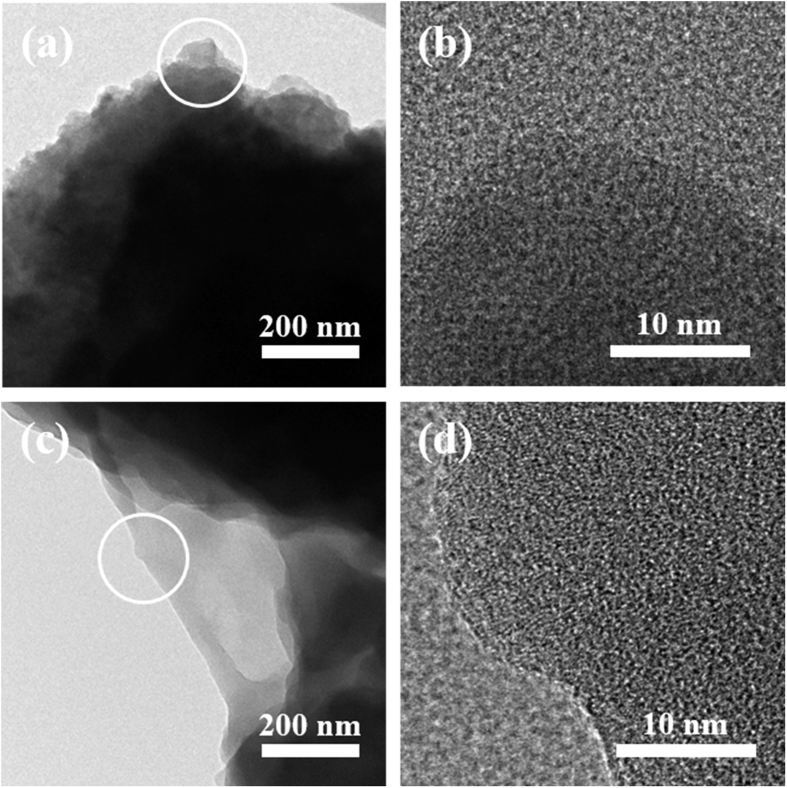
TEM images of (**a,b**) TW-Car and (**c,d**) HCl-TW-Car ((**b**) and (**d**): high-resolution TEM images of the areas circled in (**a**) and (**c**), respectively).

In order to determine the detailed microstructures of each product, TEM and high-resolution TEM images were obtained, which are shown in Fig. 4. Although sponge-like macroporous structures were seen in the SEM images of both products, transmission electron microscopy (TEM) analysis revealed critical differences in the detailed microstructures of TW-Car and HCl-TW-Car. As shown in Fig. 4b and d, unlike TW-Car, HCl-TW-Car had large quantities of micropores and mesopores (white spots in the dark gray areas) distributed within the surface of the sample, in conformance with previous studies on porous carbon structures^31^,^32^,^36^,^43^. It is important to note that such porous structures can enhance the active surface for Li^+^ storage, reduce the ion diffusion distance, and facilitate liquid electrolyte penetration in order to obtain LIB anode materials with better performance^44^.

**Figure 5:**
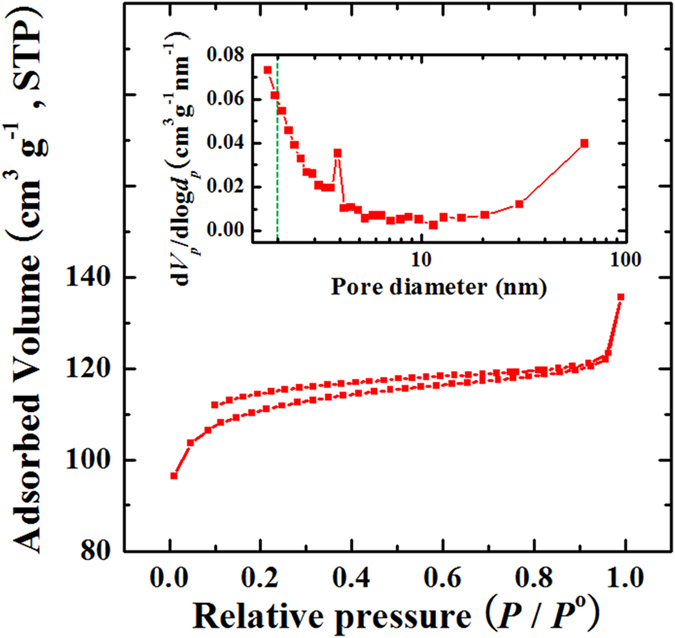
Nitrogen adsorption/desorption isotherm at 77 °C and pore size distribution of HCl-TW-Car calculated by the BJH method (the short dash line: boundary between mesopores and micropores).

In order to further analyze the detailed porosity properties of the synthesized carbon materials, nitrogen adsorption/desorption isotherm measurements at 77 K were employed and the corresponding pore size distribution curves were determined using the Barrett-Joyner-Halenda (BJH) method. As list in Table 1, it is clear that TW-Car presents a tiny amount of adsorbed N_2_ gas (the low Brunauer–Emmett–Teller (BET) surface area value, 4.99 m^2^ g^−1^) and consists of only macroporous microstructures. On the other hand, as a result of the HCl acid treatment, HCl-TW-Car exhibits a significantly increased BET surface area value (337.31 m^2^ g^−1^, Table 1) and type II/IV bimodal sorption isotherms (with type H4 hysteresis loop as classified by IUPAC) with a much higher N_2_ sorption capacity (Fig. 5), indicating that micro- (≤2 nm), meso- (2–50 nm), and macropores (≥50 nm) coexist in the material^45^. Moreover, a type H4 hysteresis loop has been associated with the presence of slit-shaped micropores located between randomly arranged graphene sheets in disordered carbon microstructures^46^. The porosities of HCl-TW-Car was also confirmed by calculating the pore size distribution, as shown in the inset of Fig. 5. The pore size distribution of HCl-TW-Car revealed the presence of some mesoporosity, with a sharp peak located at about 3.9 nm and a dramatically increasing intensity near the micropore regime and up to the macropore regime (about 62 nm), indicating hierarchically porous carbon microstructures consisting of macro-, meso-, and micropores. Additionally, as shown in Table 1, the total pore volume of HCl-TW-Car increased to 0.2098 cm^3^ g^−1^, while the average pore diameter decreased to 2.48 nm (for TW-Car, the total pore volume was 0.0071 cm^3^ g^−1^ and the average pore diameter was 5.69 nm) owing to the presence of abundant micropores. Thus, HCl-TW-Car has a hierarchically porous carbon structure with a large surface area, indicating that acid treatment before carbonization is critical to obtaining the hierarchical porous structure of the carbon product.

### Lithium electroactivity of hierarchically porous carbon electrodes

**Figure 6:**
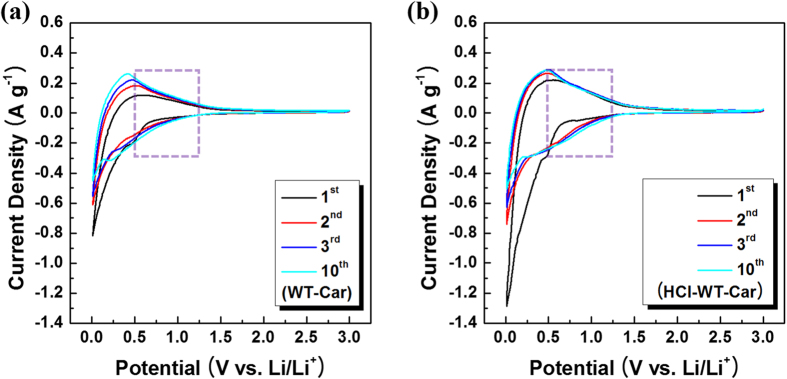
CV profiles of (**a**) TW-Car and (**b**) HCl-TW-Car (the dash boxes in (**a**) and (**b**): the hump-shaped regions).

Figure 6 shows cyclic voltammetry (CV) profiles of both TW-Car and HCl-TW-Car at a scan rate of 0.1 mV s^−1^ between 0.01 and 3.0 V. The overall shapes of the CV scans are very similar to the ones obtained by Han *et al*. using pyrolytic carbons derived from green tea biomass^30^. Both CV curves showed an irreversible cathodic peak between 0.5 and 0.6 V during the first reduction process, which occurred as a result of the decomposition of electrolyte and the formation of a solid electrolyte interface layer (SEI) on the carbon surface^25^,^47^. In subsequent cycles, the HCl-TW-Car anode showed no irreversibility, but the TW-Car anode continuously showed irreversibility under 0.6 V owing to the formation of the SEI layer. Moreover, the areas between the cathodic and anodic peaks of the HCl-TW-Car tended to be very similar during subsequent cycles, indicating that the stable SEI layers maintain cycle stability. Both products exhibited anodic peaks between 0.2 and 0.4 V and corresponding cathodic peaks at around 0 V, which can be primarily attributed to Li^+^ intercalation/deintercalation into the graphene layers^25^,^30^. We also noted a difference between the anodic and cathodic current density in the hump-shaped regions in the range of 0.5–1.25 V. Introducing acid treatment to synthesize porous carbon anode materials clearly increased this difference, which is primarily a result of adsorption/desorption on the surface or accommodation/de-accommodation in various sized pores of disordered carbon materials^48^. We conclude by CV analyses that the obtained carbon materials follow both lithiation/delithiation mechanisms, including intercalation/deintercalation and adsorption/desorption.

**Figure 7:**
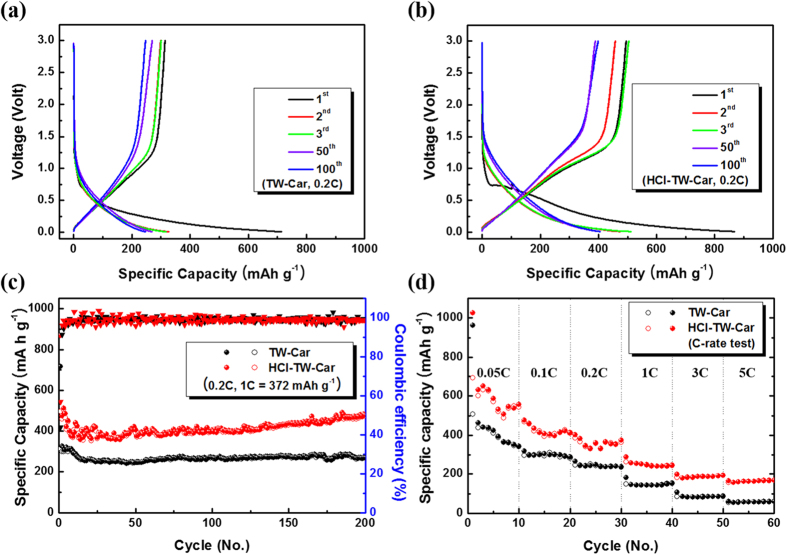
Galvanostatic charge/discharge profiles of (**a**) TW-Car and (**b**) HCl-TW-Car at 0.2C; (**c**) Cycling performance of TW-Car and HCl-TW-Car at 0.2C (1C = 372 mAh g^−1^); (**d**) rate performance of TW-Car and HCl-TW-Car at 0.05, 0.1, 0.2, 1, 3, and 5 C.

Figure 7a and b show the galvanostatic charge/discharge profiles of TW-Car and HCl-TW-Car, respectively, at a rate of 0.2 C. The first discharge curves of TW-Car and HCl-TW-Car showed much higher capacities of 715 and 869 mAh g^−1^, respectively, than the second cycle (326 and 476 mAh g^−1^, respectively). Meanwhile, the high irreversible capacities of 401 mAh g^−1^ (TW-Car) and 374 mAh g^−1^ (HCl-TW-Car) occurred in the first cycle, corresponding to initial coulombic efficiencies of 43.9% (TW-Car) and 57.0% (HCl-TW-Car). This is a well-known phenomenon seen in carbon-based electrodes resulting from SEI layer formation or irreversible Li^+^ intercalation into particular positions in highly disordered carbon materials, e.g., in the vicinity of residual hydrogen atoms, as previously reported^49^,^50^. In fact, the stable SEI layers play an important role as passivation layers that prevent the decomposition of electrolyte on the active carbon electrode. In the subsequent cycles, the galvanostatic charge/discharge curves retained their typical shapes and the irreversible capacities disappeared.

Figure 7c shows the cyclic performance of TW-Car and HCl-TW-Car at a current density of 0.2C for up to 200 cycles. After 200 cycles, HCl-TW-Car had a remarkably high reversible discharge capacity of 479 mAh g^−1^, revealing a robust cyclic performance. By contract, the TW-Car exhibited relatively low reversible discharge capacity of 270 mAh g^−1^ after 200 cycles. After the initial third cycle, the coulombic efficiencies in both products increased dramatically to the range of 96–98% and stabilized at above 98.5% for up to 200 cycles, demonstrating the outstanding reversibility of both electrodes. HCl-TW-Car always produced a much higher discharge capacity during the complete cycle, with capacities that were also higher than the theoretical capacity of general graphite (372 mAh g^−1^). Additionally, its discharge capacity of 374.5 mAh g^−1^ in the 40^th^ cycle gradually increased to 479 mAh g^−1^ after 200 cycles, with 0.653 mAh g^−1^ of capacity increase per cycle for the 40^th^ through the 200^th^ cycle on average. The same capacity increasing tendency of TW-Car electrodes is clearly seen during 500 cycles (Figure S5).

This capacity increase phenomenon has already been reported in previous studies involving rich hush-derived carbon^23^, peanut shell derived hard carbon^28^, and Portobello mushroom-derived porous carbon^47^. It can be primarily attributed to the hierarchically porous carbon structure resulting from acid treatment, suggesting that various unexposed micro- and mesopores are more accessible during repeated cycling. Another cause might be electrochemical activation of residual silicon-based compounds such as SiO_2_ after acid treatment (as shown in Fig. 3f) during repeated charge/discharge steps^51^. To examine the rate performances of TW-Car and HCl-TW-Car, the half-cells were measured by conducting charge/discharge cycles at various current densities from 0.05C to 5C each for 10 cycles, as shown in Fig. 7d. The HCl-TW-Car had reversible capacities of 556, 411, 372, 244, 192, and 168 mAh g^−1^ at 0.05, 0.1, 0.2, 1, 3, and 5C, respectively. At all current densities, HCl-TW-Car delivered higher capacities than TW-Car, which is consistent with the cycling performance at 0.2C and clearly indicates the enhanced rate capabilities of HCl-TW-Car. The enhanced rate performance of HCl-TW-Car compared to that of TW-Car is closely related to their differences in terms of BET surface area and porosity. Although hierarchically porous carbon derived from tea waste does not exhibit significantly improved electrochemical properties than several state-of-the-art heteroatom-rich biomass-derived carbon materials^32^,^34^,^52^, the HCl-TW-Car anode materials obtained in this study have various advantages, including environmental benignity, abundance, low cost of raw materials, and superior electrochemical performance than commercial graphite.

## Conclusions

In this work, we used tea waste as a raw material for preparing disordered carbon material by means of a simple, economical, and effective method involving hydrochloric acid treatment and carbonization at an optimized synthetic condition. The acid treatment applied before the carbonization process contributed to the removal of various metallic elements and further promoted the formation of hierarchically porous carbon structures, including various macro-, meso-, and micropores. Moreover, the acid treatment contributed to a tremendous increase in BET surface area in HCl-TW-Car over that in TW-Car. These unique porosity and disorder structures of HCl-TW-Car not only enhance the active surface for Li^+^ storage but also facilitate liquid electrolyte penetration and rapid transport of electrons and Li^+^. As the anode materials in LIBs, both TW-Car and HCl-TW-Car exhibit excellent cycling stability and coulombic efficiency, but HCl-TW-Car always demonstrated higher cycling performance and rate performance capacities. More impressively, HCl-TW-Car produced a capacity increase of up to 479 mAh g^−1^ after 200 cycles owing to the enhanced accessibility of unexposed micro- and mesopores and the electrochemical activation of residual silicon-based compounds.

## Methods

### Preparation of hierarchically porous carbon materials from tea wastes

The tea waste was obtained from used Sulloc brown rice green tea bags (produced by AmorePacific Co., Ltd.), which consist of brown rice (70%) and green tea (30%) harvested from South Korea. The raw materials were washed with deionized water several times and dried in a vacuum oven at 80 °C overnight. The dried tea waste was crushed to produce smaller particles of less than 250 μm to enable further processing. About 2 g crushed tea waste was refluxed with 100 ml 1.0 M HCl solution (Samchun Pure Chemical) at 120 °C in an oil bath for 2 h with constant magnetic stirring. To remove the residual acid, the product was filtered and washed with deionized water until the pH was 7, using filter paper (3 μm, HYUNDAI MICRO) and a Büchner funnel. After complete drying at 80 °C overnight, the resulting powder was transferred into a horizontal quartz tube furnace, which was purged with high-purity Ar gas for 1 h. Carbonization of the tea waste was carried out at 600 °C (heating ramp: 10 °C min^−1^) for 1 h under a high-purity Ar atmosphere (flow rate: 100 sccm). After natural cooling to room temperature, the carbonized product was ground to a fine black powder. The final hierarchically porous carbon was denoted HCl-TW-Car. For comparison, simply carbonized samples (denoted TW-Car) were also prepared following the same carbonization process without acid treatment.

### Characterization of the materials

TGA (DTG-60A, Shimadzu) was conducted from room temperature to 700 °C at a heating rate of 10 °C min^−1^ in an Ar atmosphere. XRD analysis of the products was performed using an X-ray diffractometer (SmartLab, Rigaku) with Cu Kα radiation. Raman spectra were recorded with a combined Raman FT-IR spectrometer (LabRAM ARAMIS IR2, HORIBA Jobin Yvon). The surface chemistry was investigated using X-ray photoelectron spectroscopy (XPS, X-TOOL, ULVAC). The morphologies, microstructure, and EDS of HCl-TW-Car and TW-Car samples were investigated by FE-SEM (Quanta 250 FEG, FEI) and TEM (JEM-2100F, JEOL). The BET surface area, total pore volume, and average pore diameter of the products were determined using nitrogen adsorption/desorption isotherm measurements (Tristar 3000, Micromeritics). Elemental analysis was performed using an elemental analyzer (TruSpec Micro, LECO).

### Electrochemical measurement

The electrochemical performances of the synthesized TW-Car and HCl-TW-Car anode materials were evaluated through the use of 2032 coin-type cells with lithium metal counter electrodes. To fabricate working electrodes, a mixed slurry of active materials, binder (Kynar 2801, PVdF-HFP), and conducting agent (Super P^®^, MMM carbon) were homogeneously dispersed in 1-methyl-2-pyrrolidinone (NMP, Sigma-Aldrich) with a weight ratio of 70:15:15 and pasted onto a copper foil by tape casting. To form a homogenous slurry, 1-methyl-2-pyrrolidinone (NMP, Sigma-Aldrich) was used as a dispersion medium. The tape-casted Cu foil was dried in a vacuum oven (120 °C, 5 h) and pressed using hand press under a constant pressure of 10 MPa. The fabricated coin cells consist of a working electrode (active mass 3.0 ± 0.5 mg), a counter electrode, and a separator film (Celgard 2400, Celgard) with a liquid electrolyte (1 M LiPF_6_ in ethylene carbonate (EC) and diethyl carbonate (DEC); 1:1 by volume). The assembly process of the coin cells was carried out in an Ar-filled glove box. The electrochemical characteristics of the coin cells were obtained through galvanostatic charge–discharge tests (0.2 C, C-rate) and cyclic voltammetry (between 0.01 and 3 V at a scan rate of 0.2 mV s^−1^) performed using an automatic battery cycler (MACCOR 4000, Maccor).

## Additional Information

**How to cite this article:** Choi, C. *et al*. Enhanced Lithium Storage in Hierarchically Porous Carbon Derived from Waste Tea Leaves. *Sci. Rep.* **6**, 39099; doi: 10.1038/srep39099 (2016).

**Publisher's note:** Springer Nature remains neutral with regard to jurisdictional claims in published maps and institutional affiliations.

## Supplementary Material

Supplementary Information

## Figures and Tables

**Table 1:** Detailed porosity properties of crushed TW, TW-Car, and HCl-TW-Car using the BET and the BJH methods.

Sample	BET surface area (m^2^ g^−1^)	Total pore volume (p/p_0_ = 0.988, cm^3^ g^−1^)	Average pore diameter (nm)
Crushed TW	0.07	0.0003	18.7
TW-Car	4.99	0.0071	5.69
HCl-TW-Car	337.71	0.2098	2.48
