# Supplementary material for: Enhanced Lithium Storage in Hierarchically Porous Carbon Derived from Waste Tea Leaves
Source: Sci Rep. 2016 Dec 14;6:39099. doi: 10.1038/srep39099 (PMC5155420; doi:10.1038/srep39099)
Supplement: Supplementary Information [file srep39099-s1.pdf]

Supporting Information for

Enhanced Lithium Storage in Hierarchically Porous Carbon Derived from Waste Tea Leaves

Changhoon Choi[1], Seung-Deok Seo[1], Byung-Kook Kim[2], and Dong-Wan Kim[1,*]

[1] School of Civil, Environmental and Architectural Engineering, Korea University, Seoul 136-713, Republic of Korea

[2] High-Temperature Energy Materials Research Center, Korea Institute of Science and Technology, Seoul 136-791, Republic of Korea

* Corresponding author

Prof. Dong-Wan Kim (D.–W. Kim)

Tel: +82-2-3290-4863

E-mail: dwkim1@korea.ac.kr

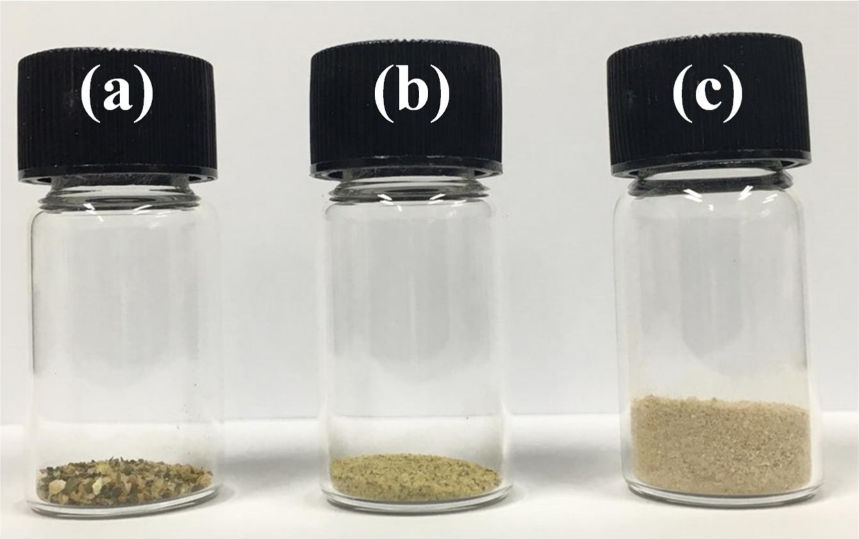

Supplementary Figure S1. Digital photograph to confirm volume change of (a) raw tea wastes, (b) crushed powder, and (c) acid-treated powder in the same weight (0.1 g).

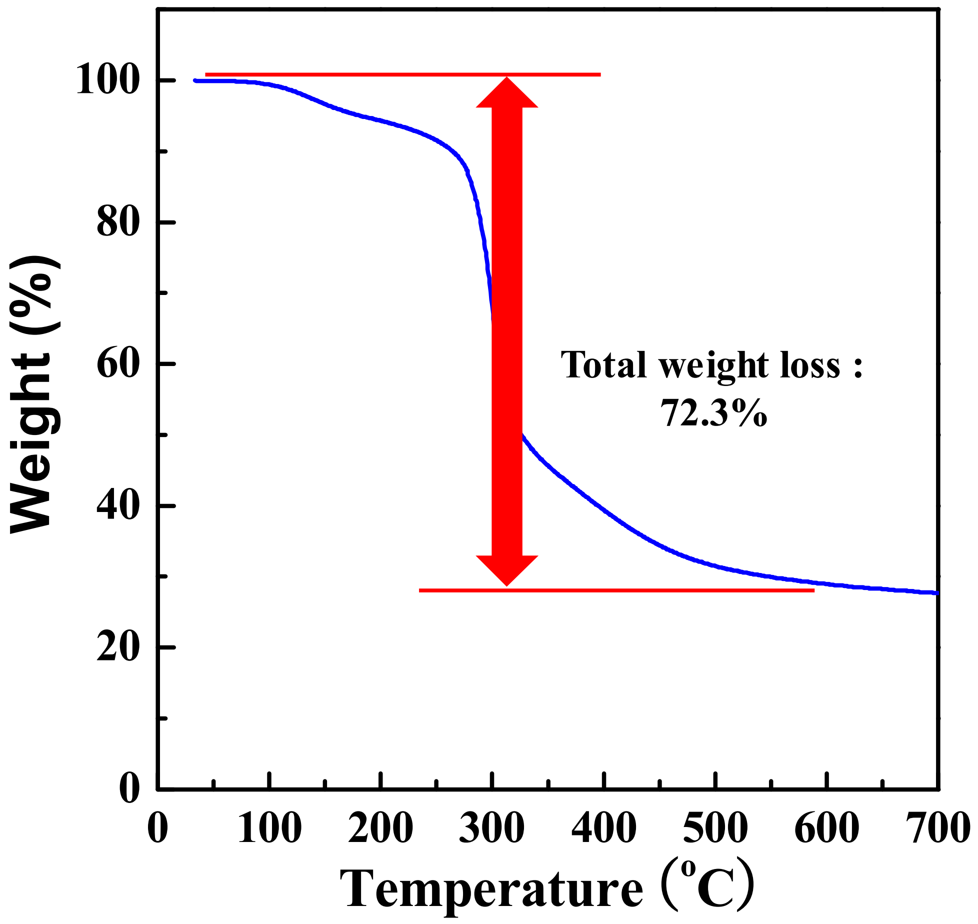

Supplementary Figure S2. TGA data of tea wastes between room temperature and 700 °C at a heating rate of 10 °C min-1 in argon atmosphere.

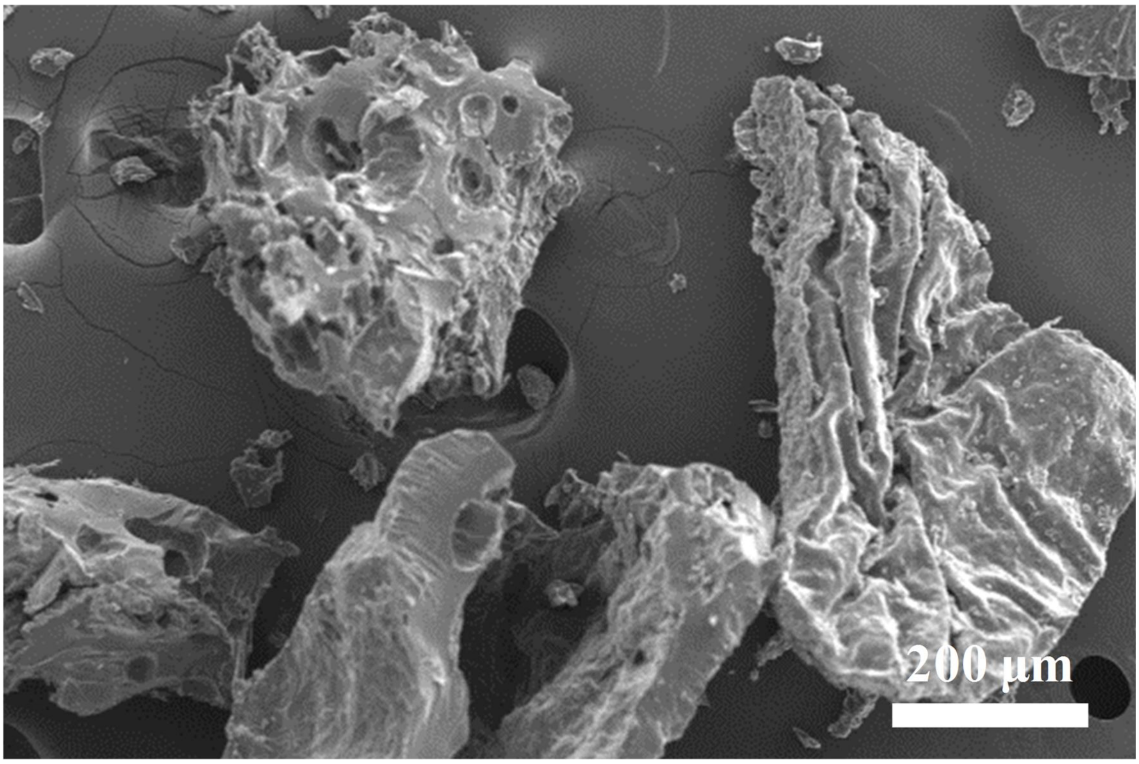

Supplementary Figure S3. Low magnification SEM image of washed tea wastes.

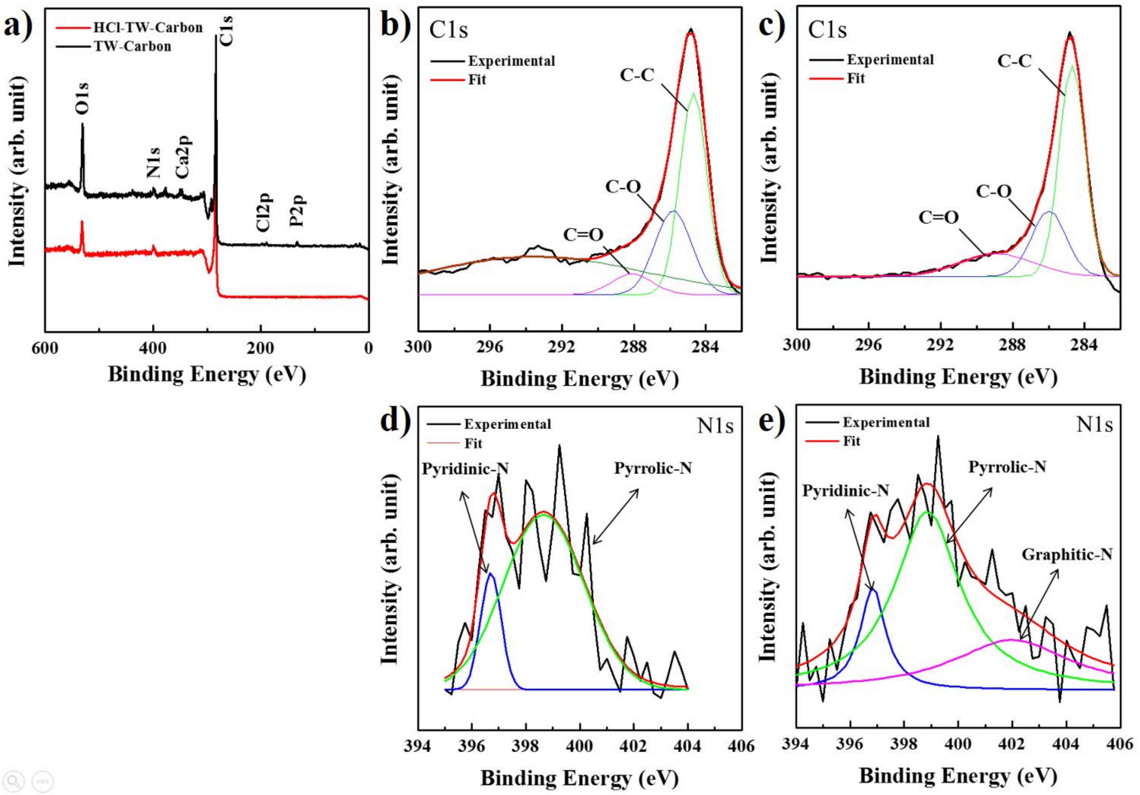

Supplementary Figure S4. a) The XPS survey spectra, b, c) C1s spectra and d, e) N1s spectra of TW-Car and HCl-TW-Car samples, respectively.

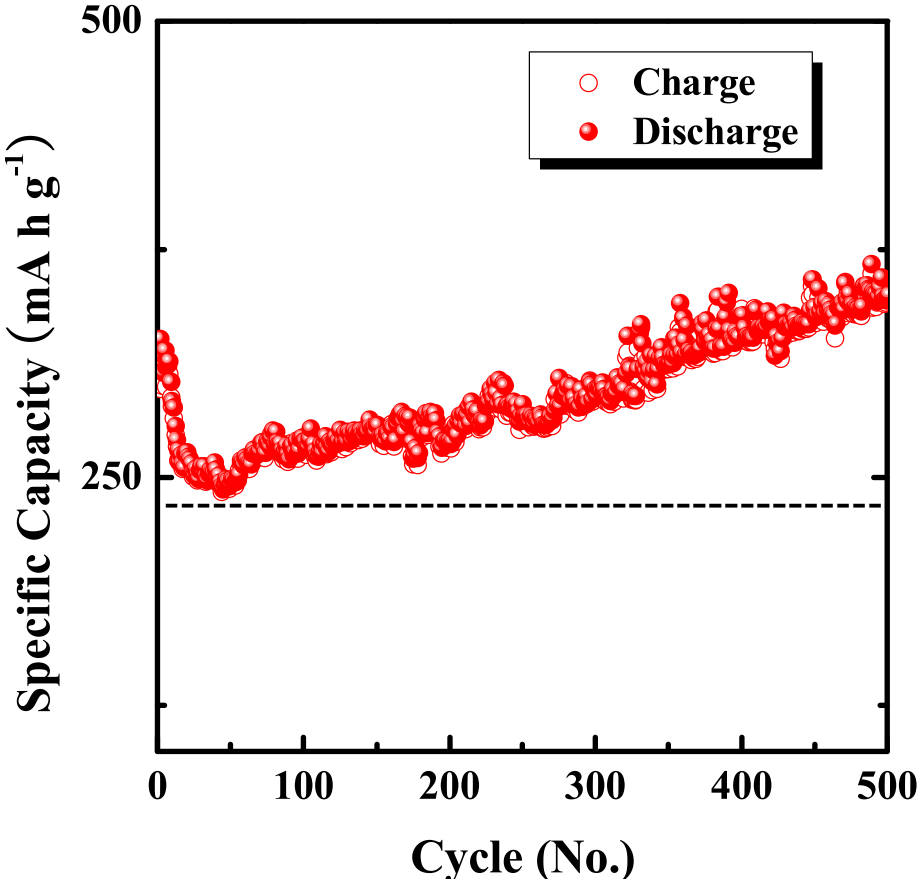

Supplementary Figure S5. Cycling performance of TW-Car at 0.2 C for 500 cycles.

Supplementary Table S1. Quantitative results of elemental ratio (wt%) by SEM-EDS analysis of TW-Car and HCl-TW-Car. (Pt*: from Pt sputtering)

**Sample**	C	O	N	S	P	K	Ca	Mg	Si	Al	Na	**Pt***
**TW-Car**	43.51	48.37	4.00	0.59	0.53	0.52	0.14	0.12	0.11	0.10	0.09	**4.81**
**HCl-TW-Car**	44.82	34.82	13.78	0.57	0.19	X	X	X	0.20	X	X	**5.62**
